# Editorial: Insights in veterinary infectious diseases: 2022

**DOI:** 10.3389/fvets.2023.1179912

**Published:** 2023-03-24

**Authors:** Mujeeb Ur Rehman

**Affiliations:** ^1^State Key Laboratory of Marine Resource Utilization in South China Sea, College of Oceanology, Hainan University, Haikou, Hainan, China; ^2^Directorate Planning and Development, Livestock and Dairy Development Department Balochistan, Quetta, Pakistan

**Keywords:** infectious diseases, host response, immunology, pathogen, genomic studies

We have entered the third decade of the twenty-first century, and the last two decades in particular of this century have already observed a series of infectious disease outbreaks in the shape of Covid-19, Ebola virus, SARS, and Zika virus, which have not only caused substantial mortality and morbidity throughout the world but also damaged peoples' socio-economic situations. At the same time, scientists have made exceptional achievements over the last few decades, leading to major advancements in the fast-growing field of infectious diseases. The biggest example of these achievements is the recent development of the COVID vaccine, which was produced in no time to save the lives of many people. In addition, the risk of these infectious diseases has helped to increase awareness among common people and governments to improve personal hygiene, sanitation, and community health care systems.

The basic goal of this special edition Research Topic was to shed light on the progress made in the past decade in the veterinary infectious diseases field and its future challenges to provide a thorough overview of the field. As such, this Research Topic solicited brief, forward-looking contributions from the editorial board members that explore the novel developments, current challenges, and latest discoveries and advancements that have been achieved as well as future perspectives in the field of veterinary infectious diseases. The issue currently includes eight papers on genome-wide association studies, host–pathogen interaction, pathogenesis, and the effect of the H9N2 vaccine on the co-infection of pathogens.

To counter the outbreaks of infectious diseases, time is required to introduce novel sampling procedures, evaluate the efficacy of diagnostic tests, and implement a hazard-specific surveillance system to cover the animal population. In a relevant area of research, in their mini review, Reggiani et al. have explained what lessons we can draw from the emergence of Omicron, the challenges in the surveillance of zoonotic diseases in wildlife, and especially the need to develop novel surveillance techniques with respect to the emergence of new infectious diseases. In addition, Liu et al. have briefly explained the sneaky features of rabies virus (RABV) with the help of available scientific results. The authors have increased our knowledge of the pathogenesis of RABV by explaining the advance steps of its progression inside the cells. The authors have provided the latest information on the cytoskeleton elements and their possible interactions with RABV.

Currently, genome-wide association studies are widely used to discover genetic markers, which could help to identify animals that are more resilient and less susceptible to a particular disease. For this purpose, genotyping and the subsequent imputation of the whole-genome sequence has allowed the identification of candidate genes, quantitative trait loci, and single-nucleotide polymorphisms in the genome associated with susceptibility to an infection. In the same area of research, Alonso-Hearn et al. have reviewed recent studies to identify the genetic markers that are less susceptible and more resilient to Johne's disease or paratuberculosis (PTB). The authors explain the associations between host genetics and PTB-associated pathology, and they enhance our knowledge on the mechanisms involved in the pathogen and disease tolerance/resistance in asymptomatic individuals. The identification of factors to tolerate the disease or to resist the infection without compromising animal health and production is of utmost importance for breeding purposes and disease control.

Over the last few years, understanding the mystery of non-expressing regions in DNA has been the key focus of researchers. Previously, these non-expressing or non-coding regions were considered as genomic garbage or DNA junk. However, the terminology of DNA junk has slowly disappeared with the advancement of comparative genomics, which has uncovered the importance of these non-coding genomic sequences in various biological processes. In the same research direction, Dong et al. have used genome-wide analysis for the detection of differentially expressed long non-coding RNAs (lncRNAs) and mRNAs in Seneca Valley virus-infected (SVV) PK15 cells. The authors have provided new insights to help understand the pathogenesis and function of the lncRNAs involved in SVV infection.

In another study, Qin et al. have evaluated the pathogenicity of multidrug-resistant *T. pyogenes* in precision-cut lung slice (PCLS) cultures from swine and in mouse models. The authors suggest porcine PCLS cultures as a 3D organ model for the study of *T. pyogenes* infection and treatment *in vitro*. Similarly, Hussain et al. have studied the necropsy, tissue samples, and intestinal contents of enterotoxemia in small ruminants from Pakistan. In addition, Mahmoud et al. have observed the effect of the H9N2 vaccine against co-infection with pathogenic *E. coli* and low pathogenic avian influenza virus (LPAIV) subtype H9N2, which is endemic in most Middle Eastern countries. The authors suggest that the LPAIV H9N2 vaccine can significantly improve the health status, amount of virus shed, and mortality rate of broiler chickens. Lastly, Ahmad et al. have reviewed the cellular and molecular processes that build disease tolerance to infection. Furthermore, they discuss how symbiotic relationships with microbes and their control by particular components of innate and adaptive immunity alter disease tolerance to infection. Altogether, this special edition Research Topic has shed light on the progress made in the past decade in the veterinary infectious diseases field and its future challenges to provide a thorough overview of the field.

## Author contributions

The author confirms being the sole contributor of this work and has approved it for publication.

